# Computational histology reveals that concomitant application of insect repellent with sunscreen impairs UV protection in an ex vivo human skin model

**DOI:** 10.1186/s13071-025-06712-3

**Published:** 2025-03-04

**Authors:** Sophie Charrasse, Titouan Poquillon, Charlotte Saint-Omer, Audrey Schunemann, Mylène Weill, Victor Racine, Abdel Aouacheria

**Affiliations:** 1 ISEM, Univ Montpellier, CNRS, IRD, Montpellier, France; 2 QuantaCell SAS, Hôpital Saint Eloi, IRMB, 80 Av Augustin Fliche, 34090 Montpellier, France; 3 EDENCOS, 39 Ancienne Route Nationale 7, 69570 Dardilly, France

**Keywords:** Insect repellent, Organelle biology, Image analysis, Sunscreen, Morphometry, Histology, Exposome, Toxicology

## Abstract

**Background:**

Histological alterations such as nuclear abnormalities are sensitive biomarkers associated with diseases, tissue injury and environmental insults. While visual inspection and human interpretation of histology images are useful for initial characterization, such low-throughput procedures suffer from inherent limitations in terms of reliability, objectivity and reproducibility. Artificial intelligence and digital morphometry offer unprecedented opportunities to quickly and accurately assess nuclear morphotypes in relation to tissue damage including skin injury.

**Methods:**

In this work, we designed NoxiScore, a pipeline providing an integrated, deep learning-based software solution for fully automated and quantitative analysis of nucleus-related features in histological sections of human skin biopsies. We used this pipeline to evaluate the efficacy and safety of three dermato-cosmetic products massively sold at the time of the study in the Montpellier area (South of France): a sunscreen containing UV filters, a mosquito repellent (with synthetic active ingredient IR3535) and a product combining a natural insect repellent plus a sunscreen. Hematoxylin and eosin or hematoxylin-eosin saffron staining was performed to assess skin structure before morphometric parameter computation.

**Results:**

We report the identification of a specific nuclear feature based on variation in texture information that can be used to assess skin tissue damage after oxidative stress or UV exposure. Our data show that application of the commercial sun cream provided efficient protection against UV effects in our ex vivo skin model, whereas application of the mosquito repellent as a single product exerted no protective or toxic effect. Notably, we found that concurrent application of the insect repellent with the sunscreen significantly decreased the UVB protective effect of the sunscreen. Last, histometric analysis of human skin biopsies from multiple donors indicates that the sunscreen-insect repellent combo displayed variable levels of protection against UV irradiation.

**Conclusions:**

To our knowledge, our study is the first to evaluate the potential toxicity of combining real-life sunscreen and insect repellent products using ex vivo human skin samples, which most closely imitate the cutaneous physiology. The NoxiScore wet-plus-dry methodology has the potential to provide information about the pharmaco-toxicological profile of topically applied formulations and may also be useful for diagnostic purposes and evaluation of the skin exposome including pesticide exposure, air pollution and water contaminants.

**Graphical Abstract:**

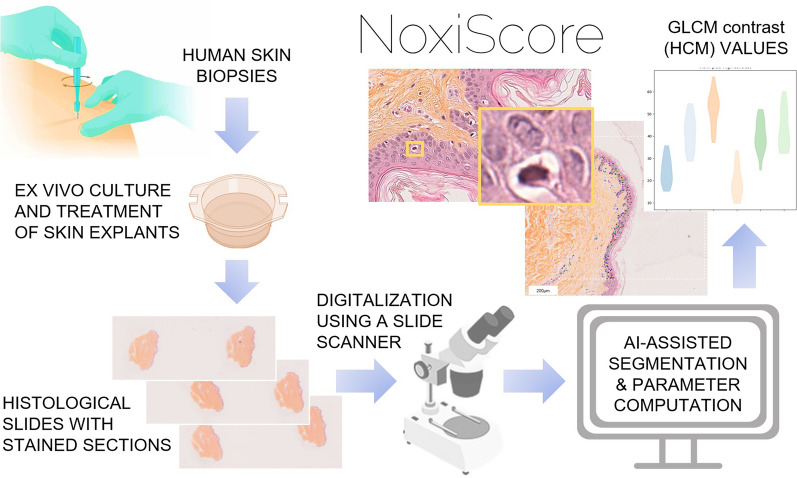

**Supplementary Information:**

The online version contains supplementary material available at 10.1186/s13071-025-06712-3.

## Background

Skin is the largest organ in the body and covers its entire external surface, acting as a biological barrier. Evolution of human skin barrier is characterized by specific genetic changes that have occurred since humans diverged from their ancestors [1] and thereafter into separate populations [2]. On a daily basis, this skin barrier is the first line of defense against physical environmental stressors [like ultraviolet (UV) radiation, blue light, high temperatures and mechanical injury], biological hazards (from bacteria, viruses, fungi, insect bites and allergens) and a wide range of environmental chemicals (including particulate matter, persistent organic pollutants, tobacco smoke, pesticides, heavy metals, etc.) [3–5].

Exposure of the human skin to foreign substances may be incidental (i.e. environmental) but can also be deliberate, such as the use of cosmetic products and pharmaceuticals. In this respect, improved awareness of skin cancers and vector-borne diseases has led to a rise in the use of sunscreens and insect repellents, especially during the summer months. Indeed, public concerns about Zika, malaria, dengue, chikungunya and West Nile virus (which are transmitted by mosquitoes), as well as Lyme borreliosis (which is spread to humans by infected ticks), have increased requests for individual protection solutions against arthropod bites [6, 7]. The World Health Organization (WHO) recommends using insect repellents as a gold standard for protection against arthropods and their associated diseases [8]. Repellents are not meant to kill insects but to keep them away to prevent bites and the spread of diseases. Insect repellent formulations can be purchased as lotions, pump sprays, aerosols or impregnated material (such as wet wipes and roll-ons). While synthetic molecules like N, N-diethyl-m-toluamide (DEET), 3-[N–n-butyl-N-acetyl] aminopropionic acid ethylester (also known as Insect Repellent 3535 or IR3535), picaridin, permethrin and N,N-diethyl phenylacetamide (DEPA) are primary ingredients in commercially available formulations [9–11], repellents of natural origin have also entered the market [such as para-menthane-3,8-diol (PMD), eucalyptus and citronella oils and other plant-derived ingredients] [12, 13]. Recently, there has been a growing demand for insect repellent products as more people are engaging in outdoor activities [14], with several mosquito species (like *Aedes albopictus*) feeding during the day. Recreational UV exposure has also dramatically increased in recent years because of outdoor activities and skin tanning for aesthetic purposes [15, 16]. Because solar UV rays are well known to cause cutaneous cancers and premature skin aging (characterized by wrinkles, loss of skin elasticity and age dyschromia) [17], health authorities recommend using sunscreens to prevent photoallergic reactions. Sunscreen formulations are generally available in lotions, gels, sprays, aerosols, suntan oils and sticks that should be applied generously to cover all exposed skin [18]. Such recent tendencies have contributed to the increase in application of sunscreens and insect repellents, both separately and combined within a single product. With climate change bringing warmer temperatures and insect outbreaks (such as in the Languedoc coast and Camargue, two areas in southern France), it is anticipated that people will increasingly incorporate these products into their daily routines.

Establishing recommendations for proper use of sunscreens and insect repellents, both separately and concurrently, is challenging [19–23] because of the wide spectrum of commercial preparations and the difficulty of finding a balance between applying effective products (i.e. products offering the greatest protection in real-life conditions of temperature, humidity, sweat and abrasion) and limiting their potential for skin irritation, dermal toxicity and systemic adverse responses. For decades, animal-based test procedures for evaluating skin and ocular irritation (such as the rabbit Draize test) were considered the gold standard for risk assessment of consumer products. However, such highly invasive tests have been criticized because of concerns about animal welfare and unnecessary use of animals and for their limited transferability to the human organism because of interspecies differences. This led to several legislative changes and implementation of alternative, animal-free toxicity testing methods [24–26]. The use of in vitro human skin models offers an interesting alternative to animal testing for the evaluation of dermato-cosmetic products like insect repellents and sunscreens [27]. While skin cell cultures and in vitro-reconstructed models may be useful for initial product testing [28–30], such systems suffer from inherent limitations in terms of cell population, viability, structure and function (including metabolism, immunity and cutaneous barrier properties) as they are not exact copies of the human skin. In contrast, ex vivo skin explants (skin biopsies) contain the full architecture of the human skin (including the epidermis, dermis, skin appendages and immune cells) and have thus emerged as a model of choice to evaluate skin toxicity and pharmacology in vitro [31–34]. In this system, tissue viability and integrity can be assessed indirectly [e.g. through the methyl thiazolyl tetrazolium (MTT) assay] or directly through histological analysis after hematoxylin and eosin (HE) staining of skin tissue explant sections [35].

The advent of computational image analysis techniques has raised the possibility that objective quantification of staining properties will gain importance in providing data for toxicological or diagnostic purposes. For instance, we recently developed a series of quantitative imaging tools to measure changes in mitochondrial morphology in stressed vs. control cells [36–38]. Likewise, any given cell type has an associated “normal” nuclear morphology. Here, we reasoned that deviations of nuclear shape can indicate tissue injury due to toxic damage to cells in the same way that nuclear abnormalities characterize certain types of diseases, such as cancer [39–46], Emery-Dreifuss muscular dystrophy [47–49], premature aging disorders like progeria syndromes [50, 51], trisomy 21 [52] and tauopathies [53]. To accurately assess nuclear and nucleus-associated spatial abnormalities, it is important to use quantitative measures of nuclear morphology instead of visual inspection and to take advantage of the new opportunities offered by artificial intelligence (AI). In this work, we developed a novel software (named NoxiScore) that uses deep learning techniques to detect nuclei in histological images of skin samples. We report the identification of a specific feature based on variation in nuclear morphometric information that can be used to assess skin tissue damage after oxidative stress, UV exposure and/or treatment with dermato-cosmetic products. Based on this novel indicator, our results indicate that, when administered concurrently, a widely used mosquito repellent can mitigate the protective effects on skin explants of a popular sun cream despite its high SPF (Sunburn Protection Factor) value.

## Methods

### Skin explant model

NativeSkin^®^ 8-mm-diameter live human skin explants (normal human skin biopsies) were purchased from Genoskin SAS (Toulouse, France). These human skin models were collected from healthy donors who underwent abdominoplasty procedures and had given informed consent. This biological material is in full compliance with the Declaration of Helsinki and all other applicable regulations. The present study is not classified as human subject research, and no Institutional Review Board approval was required. For each donor, 10 to 12 samples were generated from skin unexposed to solar radiation (with a total number of 4 donors). The NativeSkin kit, containing 12-well plates loaded with round skin biopsies and recommended culture medium, was used according to the manufacturer’s instructions. Each sample showed normal morphology and preserved skin structure, as attested by the internal quality check (histological validation) carried out by the manufacturer prior to shipping. The skin biopsies were reported lesion-free, histologically validated and virologically negative for HIV-1 and -2, hepatitis B and C and SARS-CoV-2 (see Additional file 1 for information related to skin type). NativeSkin samples can be cultured ex vivo up to 7 days at 37 °C with 5% CO_2_ and maximal humidity, without loss of tissue integrity, viability and barrier properties [54, 55]. After their delivery, the skin explants were refreshed with new medium and kept in culture prior to treatment. The culture medium was renewed every day.

### Products

Product selection was made based on a local market survey of the best-selling sunscreens, insect repellents and “combos” available for commercial distribution in the area of Montpellier (South of France). A list of items was prepared from local retail stores, including local pharmacies, supermarkets, outdoor gear shops, department stores, convenience stores and cosmetics stores. Non-topical mosquito repellent products, including those in the form of wristbands and patches, those used on clothing and outdoor gear, and area repellents such as lanterns and coils, were excluded. The survey was conducted between April 3–7, 2022. The items chosen for experimental analysis were the mosquito repellent with the commercial name “Cinq sur Cinq TROPIC 353D06-04.21” (containing IR3535 at the concentration of 35% w/w), the sunscreen “Nivea Sun sensitive “protection immediate 50 +” (containing the UV filters butyl methoxydibenzoylmethane, ethylhexyl triazone, bis-ethylhexyloxyphenol methoxyphenyl triazine) and the combo “Cinq sur Cinq Spray Citriodora FPS50” (containing the same UV filters plus plant-based extracts including *Eucalyptus citriodora* oil (hydrated, cyclized) (CAS 1245629-80-4; 10 g/100 g)). Full ingredient lists of the formulations are available as Additional file 2. The items were purchased at a local hypermarket. All the products passed full safety testing before reaching the market and were thus not assayed here for their toxicity.

### Treatments

Skin explant samples (8 mm diameter, ~ 50 mm^2^) were dried by removing the culture medium and rapidly patting them with a sterilized pad. The tested products were applied to the skin explants, and the multi-well plates were then refilled with fresh media. When applied separately as individual substances, 50 µl insect repellent or sunscreen (corresponding to ~ 0.13–0.15 mg/cm^2^) was applied to each NativeSkin sample for 30 min. In combined application, 25 µl of each substance was layered, with the sunscreen first and then the insect repellent (as recommended by the Centers for Disease Control and Prevention) [56, 57]. Dulbecco’s phosphate-buffered saline (from Gibco) was used as a negative control and treatment with 3% H_2_O_2_ (Sigma) for 24 h as a positive control of tissue damage. Protocol for H_2_0_2_ administration was adapted from [58] (see ‘Systemic administration’). For experiments involving controlled UVB irradiation, 12-well plates loaded with NativeSkin explants were placed within a BS-02 UV irradiation chamber equipped with UV-Mat dosimeter (Dr. Gröbel UV-Elektronik GmbH, Ettlingen, Germany) as previously described [36, 38]. The lamp emits UVB with a peak at 311–312 nm and partially excludes shorter wavelengths, such as UVA. Skin explants were maintained in their nourishing matrix during the irradiation process at the dose of 300 mJ/cm^2^ (irradiation time of ~ 3 min and irradiation area of the chamber being 40 × 29 cm), and fresh culture medium was added immediately after UVB exposure. To detect macroscopic defects on tissue structural integrity, post-treatment incubation duration was set at 24 h or 48 h, the classical timepoints used in similar experimental settings [59–62]. The products were also tested in conditions of exposure to real sunlight. Briefly, skin biopsies underwent one outdoor sun exposure to a high UV index (of 8) for 2 h (corresponding to ~ 144 mJ/cm^2^). The UV index was collected from Meteo France (Montpellier city, France; June 13, 2022, from 1 to 3 p.m.). Of the two skin explants that had no sunscreen or mosquito repellent, one was exposed to sunlight for 2 h (untreated skin, positive control for solar UV-induced skin damage) and one was not exposed at all (unexposed skin, i.e. negative control for solar UV-induced skin damage). Note that the single and layered products were not removed throughout the experiments.

### Histological preparation and slide scanning

At the end of the post-treatment incubation period (24 to 48 h), biopsies were fixed in 10% neutral-buffered formalin for 48 h and stored in 70% ethanol before inclusion in paraffin and slicing (at RHEM platform, Biocampus, Montpellier, France). In a first set of experiments (12 separate conditions), 42 cross-sections from a single epidermis of 3 µm thickness (10 µm apart) were prepared using a Microm HM355-S semiautomatic microtome (Thermo Scientific). In a second set of experiments (14 separate conditions), 18 cross-sections were prepared from three different donors. Hematoxylin and eosin (HE) or hematoxylin-eosin-saffron (HES) staining was performed to assess skin structure. In HE, hematoxylin stains nucleic acids, whereas eosin counter-staining is used to detect the cytoplasmic proteins. In HES, saffron highlights the dermis (by staining collagen yellow). Images were taken by the slide scanning system Hamamatsu NanoZoomer^®^ 2.0 HT (with × 40 objective, 0.23 µm/pixel, giving 180,000*90,000 pixels, 24-bit color image NDPI files), and the virtual slide system Hamamatsu NDP.view was used to observe and measure sections (MRI-INM, Montpellier, France). All slides were scanned at 20× magnification.

### Software implementation

We used the software application HistoMetriX(M) (QuantaCell, France), a computer vision solution dedicated to histology analysis coupled to a deep learning framework. A graphical user interface allows users to upload their images, assess parameters, select a quantification procedure, launch batch processing on all slides of the project and view the results. The output is made of all the segmentation masks (nuclei, cells and epidermis) and a.csv file with all the quantitative results associated with the nuclei detected in a given epidermis. The user can choose to test different analysis steps on a defined area within the image before full image quantification on the entire image. To handle the large size of images, results (masks, assemblies) were exported into a pyramidal image format using the DZI standard [63].

### AI-assisted morphometric image analysis

An automatic ROI detection algorithm is first applied to detect only the skin areas and remove the background parts of the image. The second step involves the detection of epidermis and nuclei using pretrained CNN (U-Net [64] and StarDist, [65] networks, respectively). Calibration of segmentation parameters is performed using the previsualization tool (two left images). Then, the entire set of digitized images is processed automatically on a tiled image, allowing for AI use on very large samples and at high resolution. The quantification of nuclear parameters is then performed, initially on tiled images and then merged tiles for optimal computational efficiency. The measured descriptors can be previewed before full processing in order to focus on descriptors of interest. It is possible to extract shape information (area, perimeter, aspect ratio, etc.), intensity information (mean values, variance, extreme values), texture information (GLCM contrast, GLCM energy, GLCM entropy [66]) and density information (number of neighbors, distance to the nearest neighbor) as well as, for the nuclei, their location within the epidermis. All results are then exported in.csv format. Each tile (1024 × 1024 pixels at a resolution of 20x) classically contains around 7000 nuclei. Each tissue sample takes approximately 4 min to analyze.

### NoxiScore application

NoxiScore application is a homemade application developed in Python using a graphical interface made in QT, and statistical toolboxes allow performing statistical analyses applied to different groups of tissue samples. This module loads mask results and features associated to individual detected objects (nuclei, epidermis).

### Statistics

Statistical analysis includes different statistical tools including t-test, PCA, LDA, SSMD and many data representations (charts, radars, histograms) adapted to sample analysis. This interface allows the user to define the different conditions of the samples and perform group comparisons, measurements and visualization of the effects of treatments. In the presented results, values for each descriptor are compared by the paired t-test. Significance was set as *P* < 0.05. These are tests of significance between two conditions/groups; first, an untreated skin sample is compared with a sample subjected to solar or UVB stress. Then, each sample, pretreated with a product (sunscreen and/or insect repellent), is compared with the sample subjected to UV stress. The difference between the means of the two groups analyzed is plotted on a histogram. Total number of analyzed nuclei is given in legends to Figs. 3 and 4 and Additional file 4.

### Data storage and availability

The datasets used and/or analyzed during the current study are available from the corresponding author upon reasonable request.

## Results

Histological alterations are sensitive markers that can be used to detect the noxious effects of various substances on different organs including the skin. Here, we sought to design a pipeline (called NoxiScore) for automated detection of nuclear morphology in skin histological sections and accurate quantification of nuclear abnormalities following exposure to stress or dermato-cosmetic products.

### Design of the NoxiScore workflow

**Fig. 1:**
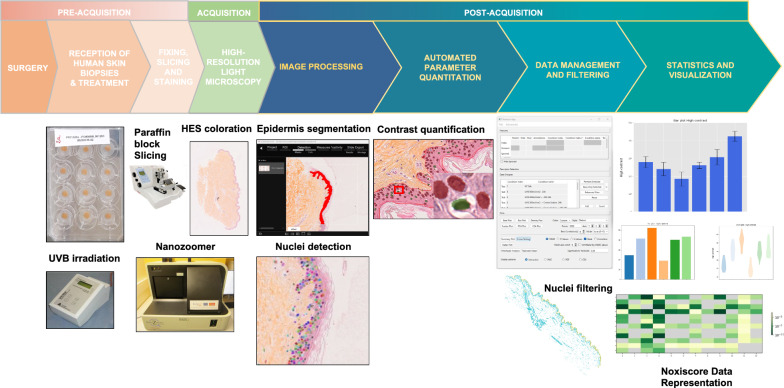
NoxiScore image analysis pipeline for nuclei analysis in histological images of ex vivo skin samples. The figure describes a new methodology for quantitative characterization of nuclear appearance in large-scale whole-slide microscopic images using human skin explants as seed material. Schematic view of the system involving pre-acquisition (treatment and preparation of the biological material), acquisition (on a modern slide scanner producing high-resolution images within minutes), and post-acquisition steps (including segmentation and feature computation). Large-scale computerized slides are taken from hematoxylin and eosin (HE) [or hematoxylin-eosin saffron (HES)] stained permanent sections of formalin-fixed and paraffin-embedded tissues. Our in-house software (NoxiScore) then handles all fundamental operations from image segmentation to statistical analysis. NoxiScore software uses deep learning during the image segmentation phase and calculates about a dozen morphological descriptors (Additional file 3). A graphical interface facilitates data collection, handling and processing

The technique used to assess the histological integrity or damage involves including ex vivo cultured skin samples in paraffin followed by coloration with hematoxylin and eosin. The bio-image analysis pipeline (Fig. 1) starts with the capture of images collected using high-resolution light microscopy of human skin samples submitted to different treatments. The NoxiScore software relies on two segmentation methods with deep learning for, respectively, epidermis and nuclei segmentation. The segmentation procedure focuses on extracting foreground objects of interest, i.e. nuclei or epidermis, from background areas. In the post-processing step, unsatisfying detected objects can be filtered out (e.g. non-epidermal nuclei are automatically removed). Segmentation is followed by computation of different categories of nuclear features, such as size, shape, intensity, texture and gradient statistics (which the human eye has difficulty perceiving). An automatic algorithm was written to compute these morphometric parameters from the produced binary segmented images. Description of the quantitative parameters generated by NoxiScore can be found in Additional file 3. Statistical analysis, as well as tables, are integrated into a user-friendly interface.

### Digital morphometric analysis of skin biopsies treated with hydrogen peroxide identifies a discriminating feature of histological alterations

**Fig. 2:**
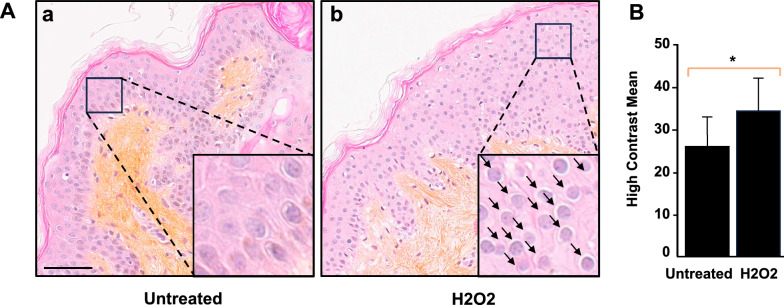
Histometric analysis of human skin biopsies treated with hydrogen peroxide. **A** Histological characterization of skin explants (8 mm diameter) exposed to hydrogen peroxide (H_2_O_2_) 24 h during ex vivo culture; 3-µm skin cross-sections of fresh human skin fixed with 10% buffered formalin and embedded in paraffin wax. Hematoxylin-eosin saffron (HES) staining. Magnification is 20× (scale bar: 50 µM). All different layers of the epidermis were detected with the purple staining of nuclei in the pink cellular background. A bright line is visible at the top of the skin that corresponds to the stratum corneum. Dermis (collagen fibers) appears in orange. Inset details show the areas of interest with haloed nuclei indicated by arrows. **B** Histograms showing high contrast mean values (expressed in percent) between untreated and H_2_O_2_-treated human skin explants with a total of 80,914 analyzed nuclei. The number of nuclei analyzed per condition is given in Additional file 4. Results are from triplicate biopsies from the same donor. Standard Student’s t-test was used to measure significance between two groups

First, we set out to identify the nuclear morphometric features significantly correlated with histological alterations in our ex vivo skin model. Skin explants were exposed to slight hydrogen peroxide (H_2_O_2_) treatment as a procedure to cause superficial oxidative stress [67–71]. The morphology of the control skin was in accordance with what is normally observed in healthy conditions, with all epidermal layers, the stratum basale, stratum spinosum, stratum granulosum and stratum corneum, appearing intact [72, 73]. Skin sections from the in vivo trial sampled at 24 h post-H_2_O_2_ exposure were compared to the control. Despite their similar overall visual appearance at the tissue level, the treated samples exhibited more condensed and darker nuclei (indicative of pyknosis) compared to the control samples (Fig. 2A). Next, we derived histograms of mean values associated with ten individual features and investigated which of these were most discriminant between the experimental conditions (Additional file 3). Noticeably, our data revealed that one of the texture parameters, namely the Gray Level Co-occurrence Matrix of contrast (GLCM contrast, herein referred to as “High Contrast Mean” or HCM), was best at discriminating between intact and H_2_O_2_-exposed skin samples (Fig. 2B). This parameter, known to be an efficient descriptor for discriminating different textural patterns [74], measures the average gray level difference between neighbor pixels (i.e. the amount of local variation between a contiguous set of pixels). In the context of our histo-morphological approach, this feature (expressed as a percentage) shows a statistically significant overabundance of nuclei surrounded by a clear halo or empty space (black arrows in Fig. 2A, panel b) in treated skin explants. Skin cells appeared partially vacuolated and disordered. Careful examination of H_2_O_2_-exposed skin sections further revealed that these nuclei enclosed within a circular or elliptical hollow area were mainly found in the stratum spinosum (the epidermal layer where desmosomes form between adjacent keratinocytes). We interpret HCM deviation from the reference situation as indicative of tissue injury due to toxic damage to cells, with the appearance of pyknotic nuclei (darker, condensed) indicating the onset of cell death processes.

### Nuclear contour based approach suggests that applying a commercial insect repellent lowers the efficacy of a popular sunscreen

**Fig. 3:**
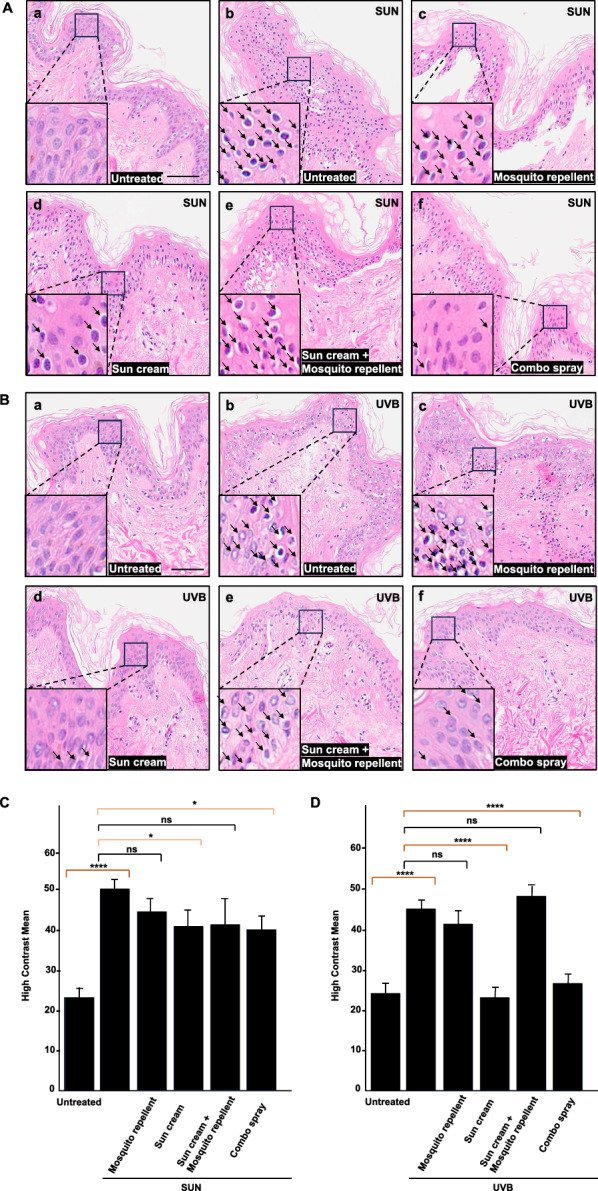
Histometric analysis of skin biopsies from a single donor treated with repellent and sunscreen formulations. Skin samples (from the same donor) were treated with (i) a sunscreen containing UV filters (panels Ad and Bd) and/or (ii) a mosquito repellent with synthetic active ingredient IR3535 (panels Ac and Bc) or (iii) a commercial preparation of a natural insect repellent plus sunscreen (‘combo’) (panels Af and Bf). The treated skin biopsies were exposed to outdoor sunlight (**A**) or to UVB irradiation in controlled conditions (**B**). Hematoxylin-eosin (HE) staining. Scale bar: 50 µM. Inset details show the areas of interest with haloed nuclei indicated by arrows. Separate treatment with the insect repellent (**c**) or the sunscreen (**d**) used as single products before sunlight exposure (2 h, UV index 8) (**A**) or UVB irradiation (**B**). Concurrent application of the insect repellent with the sunscreen. Treatment with a combination of sunscreen and insect repellent (in layers, first sunscreen and then insect repellent) (**e**). Treatment with a commercial preparation of a natural insect repellent plus sunscreen (‘combo’) (**f**). Histograms showing high contrast mean values between untreated and treated human skin explants exposed to solar radiation (**C**) or laboratory UVB irradiation (**D**). Results are from triplicate biopsies from a unique donor. Standard Student’s t-test was used to compare samples. A total of 1,185,854 nuclei were analyzed, and details of nucleus counting per condition is given in Additional file 4

Having identified a potentially relevant histological correlate of stress in ex vivo skin samples, we examined whether our pipeline could be useful in evaluating efficacy and safety of insect repellents and sunscreens administered individually or in combination. Skin samples (from the same donor) were treated with (i) a sunscreen containing UV filters and/or (ii) a mosquito repellent with synthetic active ingredient IR3535 (sunscreen first, followed by a mosquito repellent in case of combined application) or (iii) a commercial preparation of a natural insect repellent plus sunscreen (‘combo’); these three products were sold extensively at the time of the study (spring and summer 2022) in the Montpellier area (South of France). The treated skin biopsies were exposed to UVB irradiation (in the laboratory) or to sunlight (in real-life settings). Histological investigation by HE staining showed that haloed nuclei were particularly visible 24 h after sun or UVB exposure with an even distribution (Fig. 3A and B, b panels). This result suggests that UV irradiation induced alterations in the skin explants, which was confirmed using quantitative assessment of HCM values in control versus exposed samples (Fig. 3C and D; Additional file 3). As expected, this nuclear contour score was significantly reduced (Fig. 3C) and even brought to the same levels detected in unexposed skin samples (Fig. 3D) when the sunscreen was applied as a separate treatment before sunlight exposure or UVB irradiation, respectively (Fig. 3C and D). In contrast, application of the mosquito repellent as a single product did not exert any protective effect against UVB or sun exposure. Histology nicely corroborated the quantifications (panels e in Fig. 3A and B, C and D), showing that the sunscreen efficiently blocked UV radiation. Interestingly, concurrent application of the insect repellent spray with the commercial sun cream significantly reduced the UVB protective effect of the sunscreen (Fig. 3C) and even obliterated protection in case of sun exposure (Fig. 3C). Last, the sunscreen-insect repellent combo product showed moderate protection against solar radiation (Fig. 3A panel f and Fig. 3C), whereas it completely blocked the effects of UVB irradiation based on the relative absence of haloed nuclei in combination-treated skin samples (Fig. 3B panel f) and computed HCM values that were similar to unexposed samples from the assayed donor (Fig. 3D). Note that sunscreen formulations may exhibit narrow spectrum protection, covering mostly UVB and providing less photoprotection against UVA and possibly other rays of the solar spectrum.

### Distinct histological and histometric effects of individual synthetic insect repellent and sunscreen products compared to combo spray

**Fig. 4:**
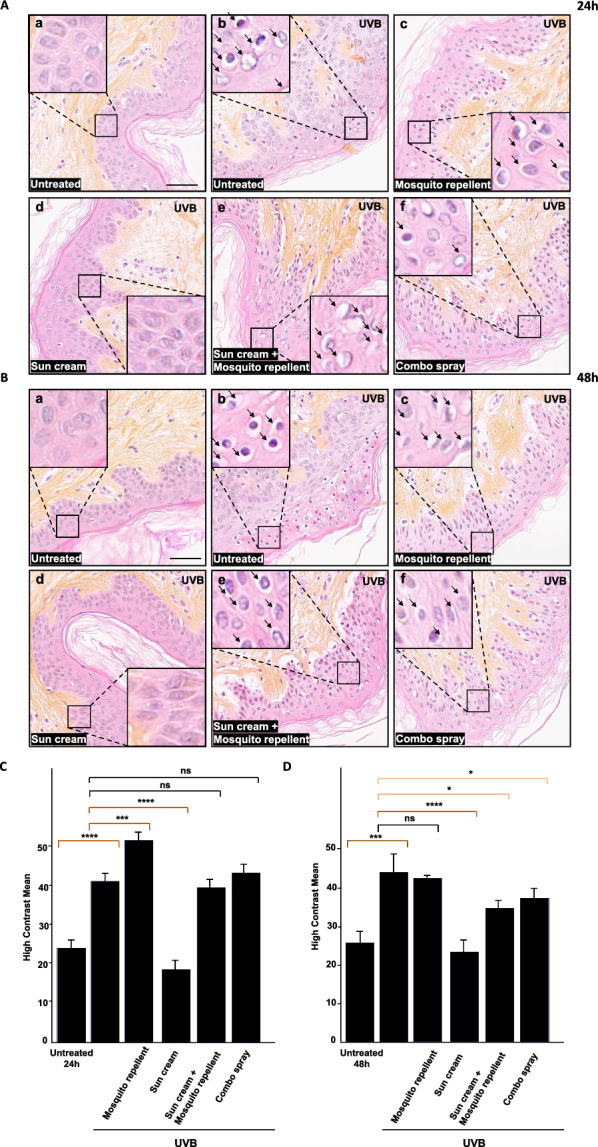
Histometric analysis of skin biopsies from multiple donors treated with repellent and sunscreen formulations. Skin samples (from the same donor) were treated with (i) a sunscreen containing UV filters and/or (ii) a mosquito repellent with synthetic active ingredient IR3535 or (iii) a commercial preparation of a natural insect repellent plus sunscreen (‘combo’). The treated skin biopsies were exposed to outdoor sunlight (**A**) or to UVB irradiation in controlled conditions (**B**). Hematoxylin-eosin saffron (HES) staining. Scale bar: 50 µM. Inset details show the areas of interest with haloed nuclei indicated by arrows. Separate treatment with the insect repellent (**c**) or the sunscreen (**d**) used as single products before sunlight exposure (UV index 8) or UVB irradiation (300 mJ/cm^2^). Concurrent application (**e**) of the insect repellent with the sunscreen. Treatment with a combination of sunscreen and insect repellent (in layers, first sunscreen and then insect repellent) (**e**). Treatment with a commercial preparation of a natural insect repellent plus sunscreen (‘combo’) (**f**). Histograms showing high contrast mean values between untreated and treated human skin explants exposed to laboratory UVB irradiation for 24 h (**C**) or 48 h (**D**). Results are from triplicate biopsies from three different donors. Standard Student’s t-test was used to compare samples. A total of 725,397 nuclei were analyzed, and details of nuclei counting per condition are given in Additional file 4

In a third set of experiments, the number of donors was raised to *N* = 3, and histological analysis was conducted by HES (to better separate the epidermis from the dermis) at both 24 h and 48 h after UVB irradiation. Agreeing with the results described in the previous section, UVB exposure had a significant impact on the detection of haloed nuclei (Fig. 4A and B, b panels), the proportion of which doubled post-UVB irradiation based on HCM quantification (Fig. 4C and D). Cells in the deep epidermal layers appeared partially vacuolated and irregularly arranged. Application of the sunscreen as a single product provided complete protection against UVB effects (Fig. 4A and B, d panels and Fig. 4C and D), whereas separate application of the insect repellent enhanced the effects of UVB exposure (evaluated 24 h post-irradiation) or tended to be neutral against UVB treatment (at 48 h) (Fig. 4A and B, c panels and Fig. 4C and D). Here again, concomitant application of the insect repellent with the sunscreen impaired or significantly diminished its protective effect against UVB (Fig. 4A and B, e panels and Fig. 4C and D). Contrary to the previous experiment, the results indicate that the sunscreen-insect repellent combo had a null (Fig. 4A panel f, at 24 h post-UVB irradiation) or limited impact (Fig. 4B panel f, 48 h) on the number of haloed nuclei and related HCM parameter values (Fig. 4C and D). This inter-individual heterogeneity in the responses may be linked to donor-to-donor variability in skin explants.

## Discussion

In recent years, interest has grown in computer-assisted morphometry to investigate the cellular and nuclear changes correlated with the physio-pathological status of histologically stained sections [39–53]. Artificial intelligence has also moved to the forefront of digital histology, bringing opportunities in terms of reliability, objectivity and reproducibility [75, 76]. At the nuclear level, morphological phenotypes were usually qualitatively described through visual clues as ‘abnormal’ or ‘dysmorphic,’ which was low throughput and often subjected to observer bias. Quantitative, feature-based approaches now allow for quick analysis of large sets of imaging data through an automated process and can extend the scope of descriptive features beyond those readily perceived by the human eye [77]. In the present study, we developed NoxiScore, a pipeline providing an integrated, deep learning-based software solution for fully automated and quantitative analysis of nuclear shape in HE-stained images of human skin biopsies. A number of easy-to-compute features can reveal the presence of nuclear singularities. These include a novel indicator herein named HCM (for high contrast mean) that relates to the haloed shape of nuclei in UV-exposed epidermal regions of human skin cross-sections. Such nuclei surrounded by a clear empty space could be seen in various publications dealing with skin histology (see for instance Fig. 2 in Han et al. [23]). While already known as a texture feature [78–80], most programs and studies were interested in analyzing the nucleus per se and not its surrounding space, which probably explains why deviations in this specific parameter went largely unnoticed in previous studies. Our wet-plus-dry technology based on computational histology of human skin explants may be useful for drug screening purposes, safety testing, evaluating health risks associated with exposure to environmental chemicals, the pharmaco-toxicological assessment of active ingredients and formulations and cell cultures and reconstructed skin models. To examine the effects of various test substances on the epidermis, viability assays [e.g. MTT or lactate dehydrogenase (LDH) assays] and immunohistochemical (IHC) staining are classically performed. However, these techniques are very expensive and time-consuming, especially when conducted for many specific biomarkers. Future research is needed to determine whether quantification of HCM values could represent a relevant and convenient biomarker that changes primarily with cellular injury in the ex vivo human skin model. To this end, correlation between HCM values and the percentage of pyknotic nuclei (often characterized by their condensed, shrunken, irregular, non-spherical shape and darker shade) or other cell death indicators (e.g. caspase-3 activation in the case of apoptosis evaluation) will have to be formally investigated in future studies. Evaluation of a greater number of donors will be essential to determine the robustness of this parameter (i.e. sensitivity and specificity) prior to its establishment as a formal indicator of epidermal damage. Notably, although we presented our histometric framework with application to skin explants, it could potentially be tailored to a broad scope of tissues since many classification schemes rely on nuclear feature analysis.

Insect repellent is one of the major preventive methods against mosquito-borne diseases, while sunscreens help prevent sunburns and skin cancers. These products are particularly crucial for travelers and other people who engage in outdoor activities and are exposed to both mosquito stings and solar radiation. People who engage in outdoor activities and those who live in sunny areas with many insects including mosquitoes (such as in the Languedoc coastal region and near Camargue, southern France) are often tempted to overuse these products, especially on hotter or windier days. Formulations containing different active ingredients in various concentrations are available on the market. Sunscreens and insect repellents are often used together and combined for ease of use in some commercial ‘combo’ products. However, toxicity information on combination use is currently limited, although there are research studies on efficacy testing. The observations presented here suggest that, based on histological analysis and HCM scores, dual application of a popular synthetic insect repellent (containing IR3535) with a widely distributed commercial sunscreen induced a significant decrease in the protective effects against UV exposure. A similar finding was reported for DEET (N,N-diethyl-meta-toluamide), which was found to hinder the efficacy of sunscreen when used concurrently [21, 81] (in addition to being a skin irritant [23]). Here, addition of an insect repellent containing botanical extracts to the sunscreen formulation led to heterogeneous results in terms of photoprotection, probably due to inter-donor variations. Plant-based insect repellents have been reported to be less effective with shorter durations of protection compared to their synthetic equivalents, and some may be noxious if used for longer duration [82, 83]. At the same time, co-administration of a synthetic insect repellent like DEET together with other chemical substances, such as the sunscreen compound oxybenzone [19, 20, 84, 85], is suspected to increase absorption of all topically applied products (including pesticides [86]) and to pose a health risk. For topical preparations containing combinations of natural or synthetic repellents with sunscreen, the frequency of application may also be an issue because insect repellents should be reapplied less often than sunscreens [87]. Note that absolute evaluation of these results is difficult since the final products contain small amounts of ingredients along with many other ingredients (Additional file 2). It will also be necessary to implement other application protocols, such as applying mixtures of products instead of layering, to confirm our conclusions. Last, certain products may prove to be toxic in the ex vivo model but non-toxic in vivo, or ex vivo findings may overestimate the direct effects expected in vivo. Although skin explants give a better representation of the in vivo milieu and cell composition than in vitro cultured cells, understanding the specific limitations of our ex vivo pipeline, combining it with in vivo studies when feasible and considering the potential impact of the application protocols, may help devise more advanced future studies.

## Conclusions

To our knowledge, our study is the first to evaluate the potential toxicity of combining real-life sunscreen and insect repellent formulations in an ex vivo human skin model, which provides the closest representation of the cutaneous physiology. The described methodology has the potential to inform health policy guidelines and end users on how to chose effective and safe insect repellents and sunscreens from the vast spectrum of commercially available products.

## Supplementary Information

Additional file 1. Information on biological material. Synthesis of the information displayed on the Genoskin batch release certificates.

Additional file 2. Ingredient lists of the products used in the study.

Additional file 3. Histograms showing mean values of the different morphometric parameters computed by NoxiScore. Skin samples (from the same donor) were treated or not (first two lines of each histogram; two biopsies from the same patient) with a sunscreen containing UV filters (line 4 of each histogram) or a mosquito repellent with synthetic active ingredient IR3535 (line 5 of each histogram) 30 min before exposure to UVB irradiation in controlled conditions (lines 3, 4 and 5 of each histogram). Histograms showing quantification of nuclei parameters including area, perimeter, roundness, intensities maximum, minimum and mean, GLCM contrast, energy, entropy and homogeneity 24 h after UVB irradiation. Results are from triplicate biopsies from a unique donor. Standard t-test was used to compare samples.

Additional file 4. Nuclei counting and parameter quantification per condition. The number of nuclei shows the number of cells analyzed.

## Data Availability

The data that support the findings of this study are available on request from the corresponding author. Software for this research is not publicly available due to possible future commercial exploitation.
