# Supplementary material for: Computational histology reveals that concomitant application of insect repellent with sunscreen impairs UV protection in an ex vivo human skin model
Source: Parasit Vectors. 2025 Mar 4;18:84. doi: 10.1186/s13071-025-06712-3 (PMC11881410; doi:10.1186/s13071-025-06712-3)
Supplement: Supplementary file 1 — Additional file 1. Information on biological material. Synthesis of the information displayed on the Genoskin batch release certificates. [file 13071_2025_6712_MOESM1_ESM.pdf]

| Patient | Sex | Age | Fitzpatrick classification | Skin desease | Medication | Anatomical site |
|---|---|---|---|---|---|---|
| 1 | Female | 37 | 2 | None | None | Abdomen |
| 2 | Female | 47 | 2 | None | Cetirizine | Abdomen |
| 3 | Female | 32 | 3 | None | None | Abdomen |
| 4 | Female | 31 | 2 | None | Ventoline | Abdomen |
