# Supplementary material for: Computational histology reveals that concomitant application of insect repellent with sunscreen impairs UV protection in an ex vivo human skin model
Source: Parasit Vectors. 2025 Mar 4;18:84. doi: 10.1186/s13071-025-06712-3 (PMC11881410; doi:10.1186/s13071-025-06712-3)
Supplement: Supplementary file 2 — Additional file 2. Ingredient lists of the products used in the study. [file 13071_2025_6712_MOESM2_ESM.pdf]

Product Name	Composition
**Nivea Sun sensitive protection immediate" 50+ (sun cream)**	Aqua, Alcohol Denat., Butyl Methoxydibenzoylmethane, Ethylhexyl Triazone, Bis-Ethylhexyloxyphenol Methoxyphenyl Triazine, Isopropyl Palmitate, C12-15 Alkyl Benzoate, Dibutyl Adipate, Butylene Glycol Dicaprylate/Dicaprate, Diethylamino Hydroxybenzoyl Hexyl Benzoate, Glyceryl Stearate, Phenylbenzimidazole Sulfonic Acid, Glycerin, Glycyrrhiza Inflata Root Extract, Aloe Barbadensis Leaf Juice Powder, Tocopheryl Acetate, Hydroxypropyl Starch Phosphate, Microcrystalline Cellulose, C18-38 Alkyl Hydroxystearoyl Stearate, Copernicia Cerifera Cera, Hydrogenated Rapeseed Oil, Silica Dimethyl Silylate, Cetyl Palmitate, Xanthan Gum, Sodium Stearoyl Glutamate, Ethylhexylglycerin, Caprylyl Glycol, Trisodium EDTA, Cellulose Gum, Sodium Hydroxide, Sodium Chloride. Active ingredients : Licorice Extract, Bio Aloe Vera
**Cinq sur Cinq TROPIC 353D06-04.21 (insect repellent)**	Ethyl Butylacetylaminopropionate (Insect Repellent 3535) (25%), Alcohol Denat., Aqua, PEG-7 Glyceryl Cocoate, Octyl Methoxycinnamate, Hexyl Cinnamal, Benzyl Salicylate, Limonene, Linalool, Citronellol, Butylphenyl Methylpropional, Geraniol, Amyl Cinnamal, Citral, Alpha-Isomethyl Ionone, Benzyl Benzoate, Parfum (Fragrance)
**Cinq sur Cinq Spray Citriodora FPS50 (combo)**	Aqua, Alcohol Denat., Butyl Methoxydibenzoylmethane, Ethylhexyl Triazone, Bis-Ethylhexyloxyphenol Methoxyphenyl Triazine, Isopropyl Palmitate, C12-15 Alkyl Benzoate, Dibutyl Adipate, Butylene Glycol Dicaprylate/Dicaprate, Diethylamino Hydroxybenzoyl Hexyl Benzoate, Glyceryl Stearate, Glycerin, Phenylbenzimidazole Sulfonic Acid, Glycyrrhiza Inflata Root Extract, Aloe Barbadensis Leaf Juice Powder, Tocopheryl Acetate, Microcrystalline Cellulose, Cellulose Gum, C18-38 Alkyl Hydroxystearoyl Stearate, Copernicia Cerifera Cera, Hydrogenated Rapeseed Oil, Cetyl Palmitate, Xanthan Gum, Sodium Stearoyl Glutamate, Sodium Hydroxide, Sodium Chloride, Caprylyl Glycol, Ethylhexylglycerin, Trisodium EDTA
