# Supplementary figures and images for: Computational histology reveals that concomitant application of insect repellent with sunscreen impairs UV protection in an ex vivo human skin model
Source: Parasit Vectors. 2025 Mar 4;18:84. doi: 10.1186/s13071-025-06712-3 (PMC11881410; doi:10.1186/s13071-025-06712-3)

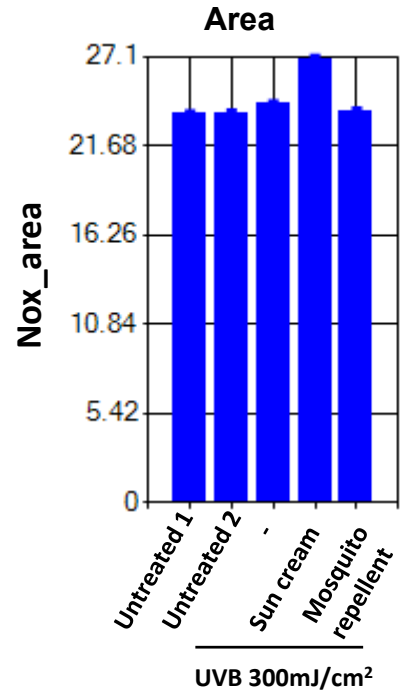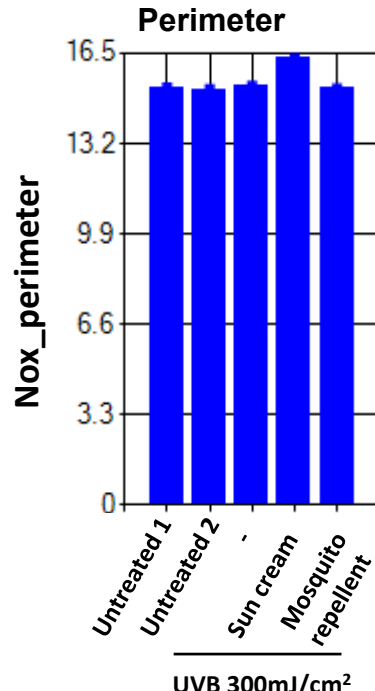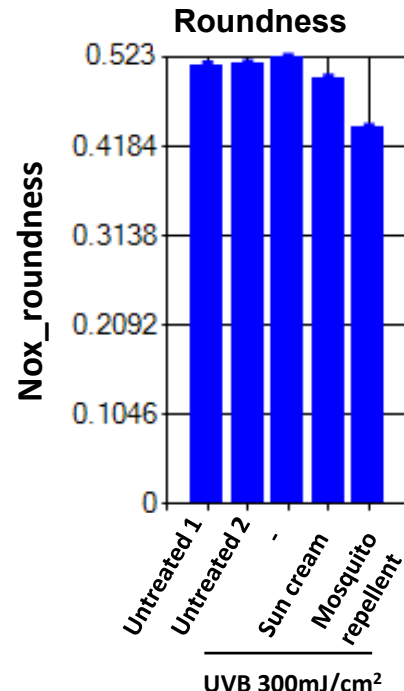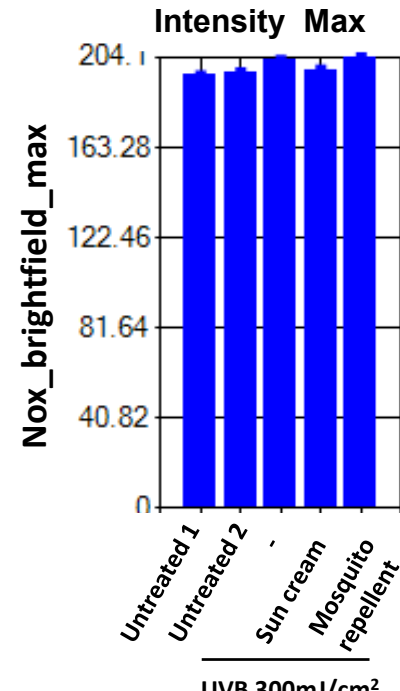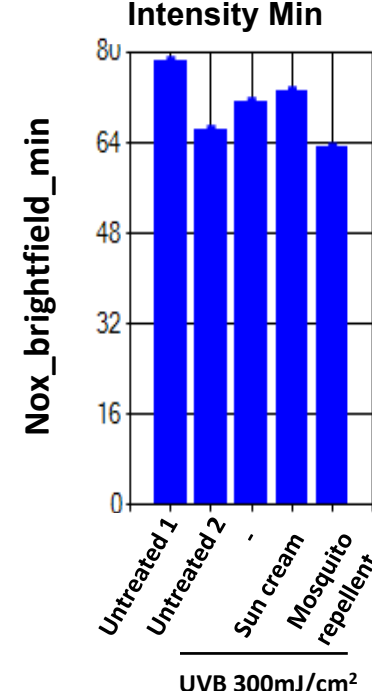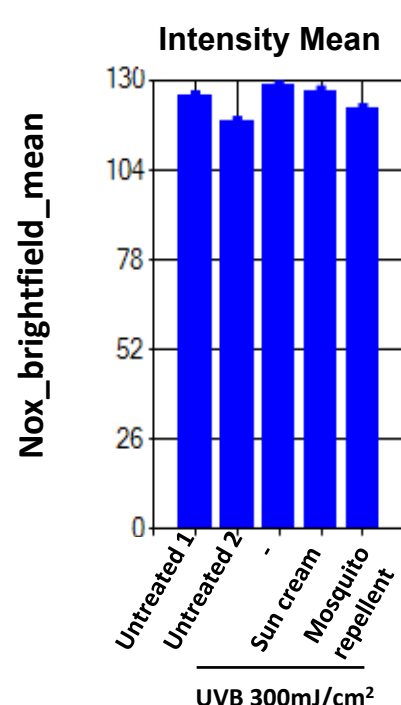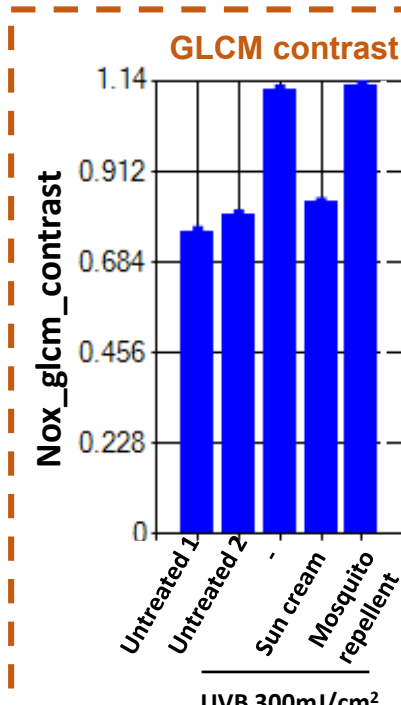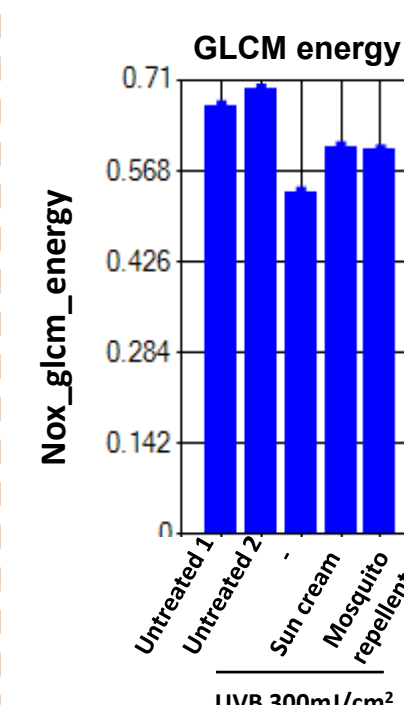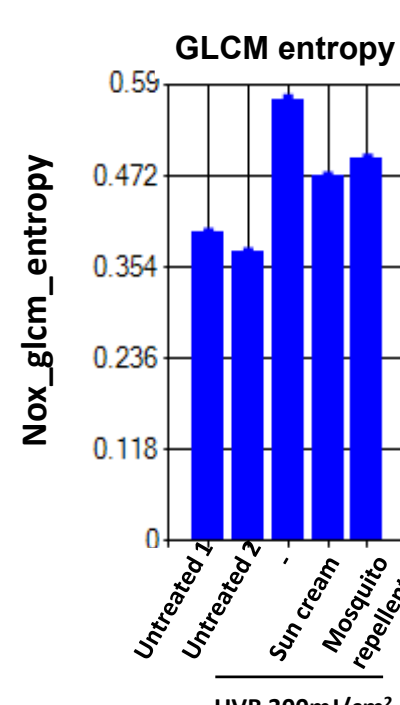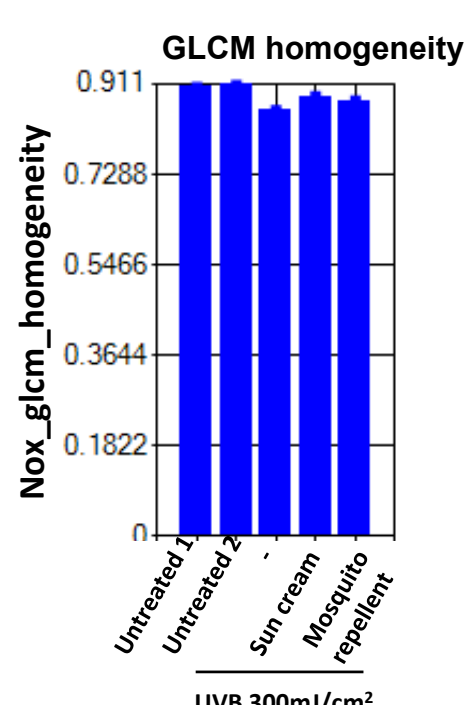

Supplement: Supplementary file 3 — Additional file 3. Histograms showing mean values of the different morphometric parameters computed by NoxiScore. Skin samples (from the same donor) were treated or not (first two lines of each histogram; two biopsies from the same patient) with a sunscreen containing UV filters (line 4 of each histogram) or a mosquito repellent with synthetic active ingredient IR3535 (line 5 of each histogram) 30 min before exposure to UVB irradiation in controlled conditions (lines 3, 4 and 5 of each histogram). Histograms showing quantification of nuclei parameters including area, perimeter, roundness, intensities maximum, minimum and mean, GLCM contrast, energy, entropy and homogeneity 24 h after UVB irradiation. Results are from triplicate biopsies from a unique donor. Standard t-test was used to compare samples. [file 13071_2025_6712_MOESM3_ESM.pdf]
