# Supplementary material for: Computational histology reveals that concomitant application of insect repellent with sunscreen impairs UV protection in an ex vivo human skin model
Source: Parasit Vectors. 2025 Mar 4;18:84. doi: 10.1186/s13071-025-06712-3 (PMC11881410; doi:10.1186/s13071-025-06712-3)
Supplement: Supplementary file 4 — Additional file 4. Nuclei counting and parameter quantification per condition. The number of nuclei shows the number of cells analyzed. [file 13071_2025_6712_MOESM4_ESM.pdf]

**Figure**	**Condition**	**Treatment**	**Nuclei Count**
Fig. 2	24h	Untreated	56667
	24h	H2O2	24247
Fig. 3	24h	Untreated	43695
	SUN 24h	Untreated	89155
	SUN 24h	Mosquito repellent	86095
	SUN 24h	Sun cream	126724
	SUN 24h	Sun cream + Mosquito repellent	58268
	SUN 24h	Combo spray	76451
	24h	Untreated	116182
	UVB 24h	Untreated	195586
	UVB 24h	Mosquito repellent	98585
	UVB 24h	Sun cream	142366
	UVB 24h	Sun cream + Mosquito repellent	64272
	UVB 24h	Combo spray	132170
Fig. 4	24h	Untreated	68061
	UVB 24h	Untreated	53094
	UVB 24h	Mosquito repellent	61927
	UVB 24h	Sun cream	74989
	UVB 24h	Sun cream + Mosquito repellent	61669
	UVB 24h	Combo spray	48599
	UVB 48h	Untreated	56667
	UVB 48h	Untreated	59803
	UVB 48h	Mosquito repellent	58285
	UVB 48h	Sun cream	65065
	UVB 48h	Sun cream + Mosquito repellent	57044
	UVB 48h	Combo spray	60194
